# Supplementary material for: Bias and Precision of Parameter Estimates from Models Using Polygenic Scores to Estimate Environmental and Genetic Parental Influences
Source: Behav Genet. 2020 Dec 10;51(3):279–88. doi: 10.1007/s10519-020-10033-9 (PMC8093160; doi:10.1007/s10519-020-10033-9)
Supplement: Supplementary file 1 — Electronic supplementary material 1 (PDF 2299 kb) [file 10519_2020_10033_MOESM1_ESM.pdf]

(Supplementary Notes) Vertical transmission estimation via structural equation modeling (VT-SEM)

Yongkang Kim[1,*], Jared V. Balbona[1], Matthew C. Keller[1,2,*]

1 Institute for Behavioral Genetics, University of Colorado at Boulder

2 Department of Psychology & Neuroscience, University of Colorado at Boulder

***yongkangkim87@gmail.com *matthew.c.keller@gmail.com**

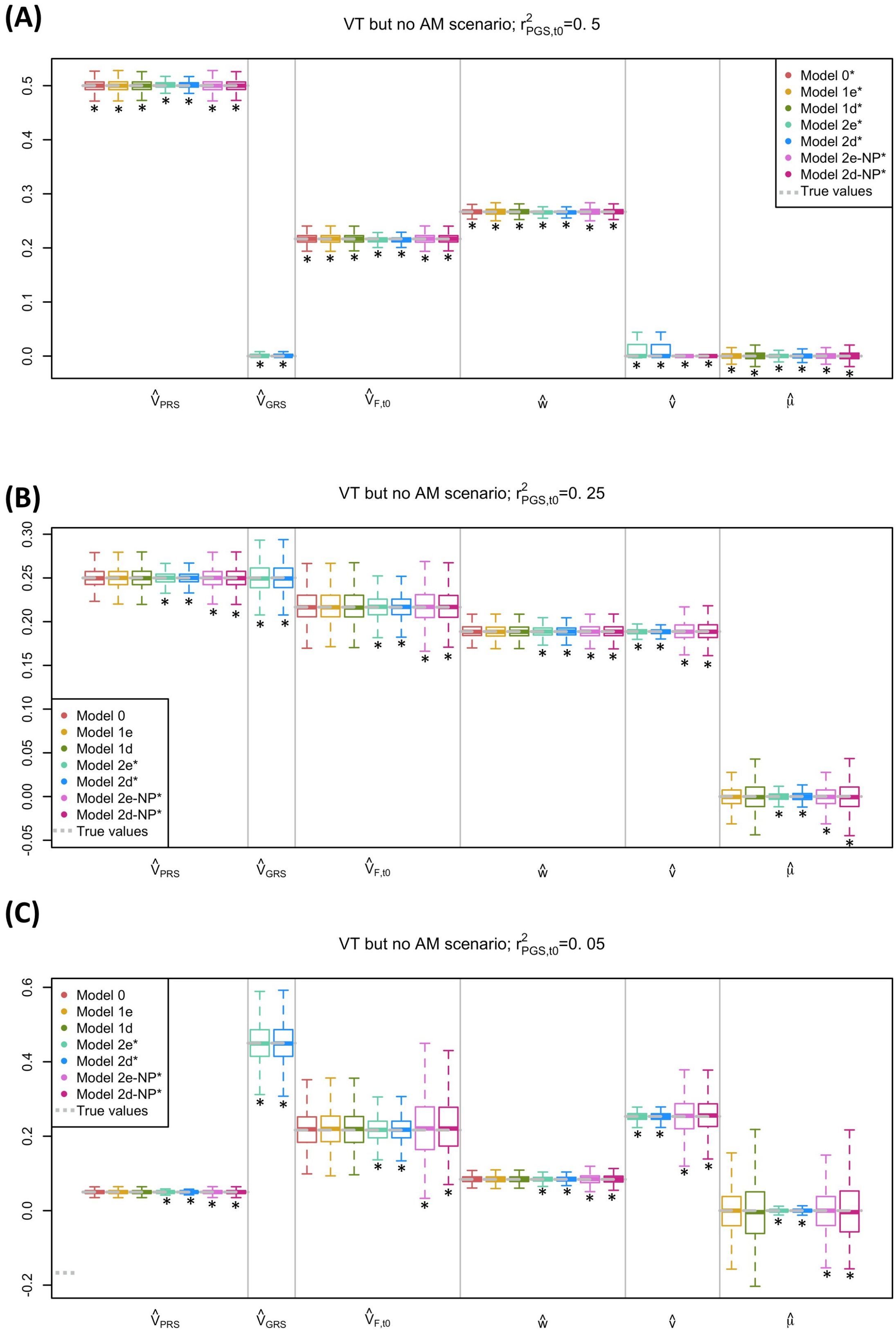

*Model for which assumptions were met

Suppl Figure 1. Comparison of estimates across models when there is VT but no AM. For each simulation, $h^2_{t0} = .50$, $r_{mate} = 0$, $V_{F,t0} = .15$, and $n_{fam} = 16K$. **(A)** $r^2_{PGS,t0} = .50$. **(B)** $r^2_{PGS,t0} = .25$. **(C)** $r^2_{PGS,t0} = .05$. Boxplots show first quartile, median, and third quartile of estimates, with whiskers at the 2.5% and 97.5% quantiles. Equilibrium values of parameters are grey dashed lines. $*$ Models where assumptions about AM and $r^2_{PGS,t0}$ are met.

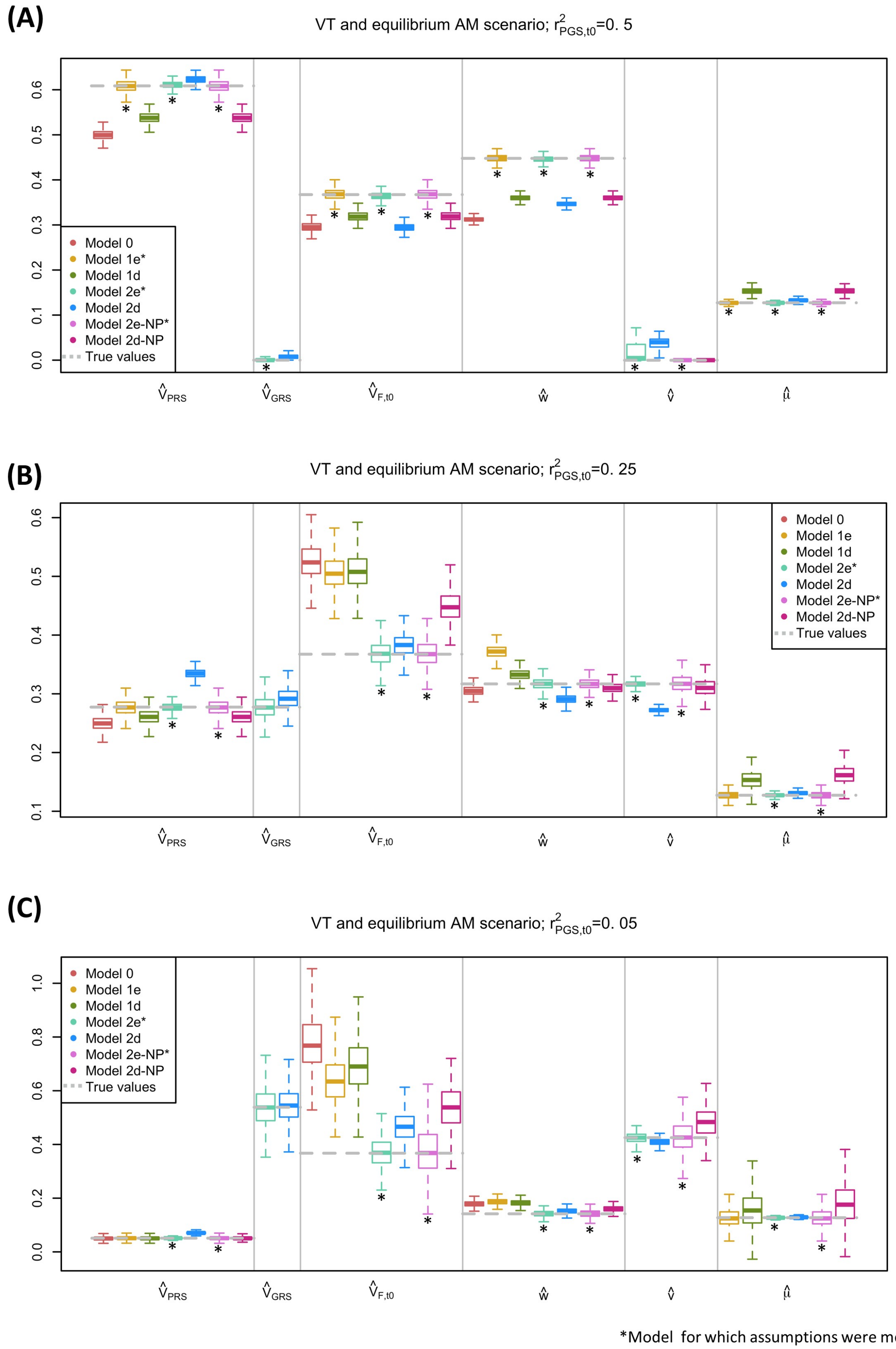

Suppl Figure 2. Comparison of estimates across models when there is VT and equilibrium AM. For each simulation, $h^2_{t0} = .50$, $r_{mate} = .25$, $V_{F,t0} = .15$, and $n_{fam} = 16K$. **(A)** $r^2_{PGS,t0} = .50$. **(B)** $r^2_{PGS,t0} = .25$. **(C)** $r^2_{PGS,t0} = .05$. See **Figure ??** note for additional details.

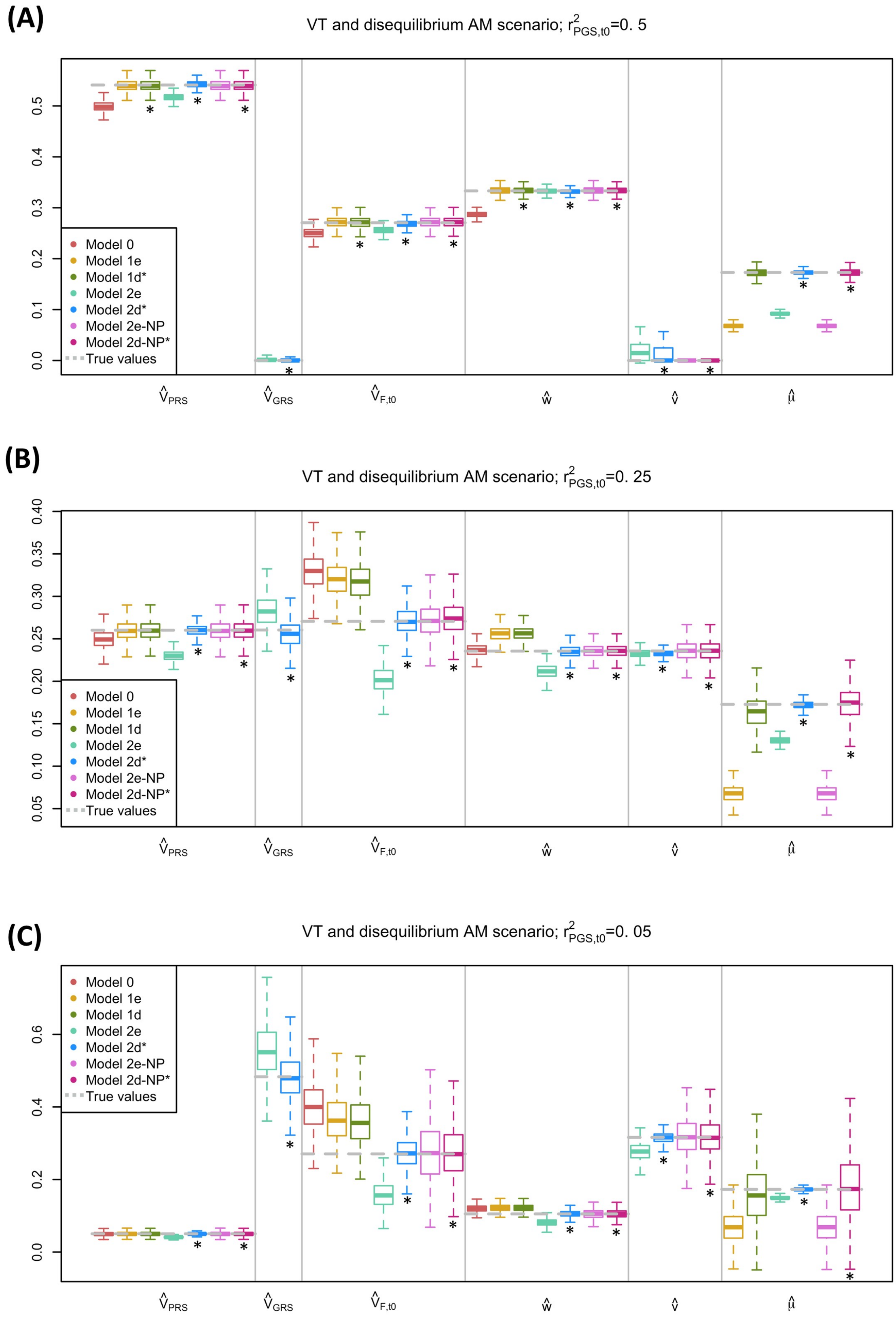

*Model for which assumptions were met

Suppl Figure 3. Comparison of estimates across models when there is VT and disequilibrium AM. For each simulation, $h^2_{t0} = .50$, $r_{mate} = .25$, $V_{F,t0} = .15$, and $n_{fam} = 16K$. **(A)** $r^2_{PGS,t0} = .50$. **(B)** $r^2_{PGS,t0} = .25$. **(C)** $r^2_{PGS,t0} = .05$. See **Figure ??** note for additional details.

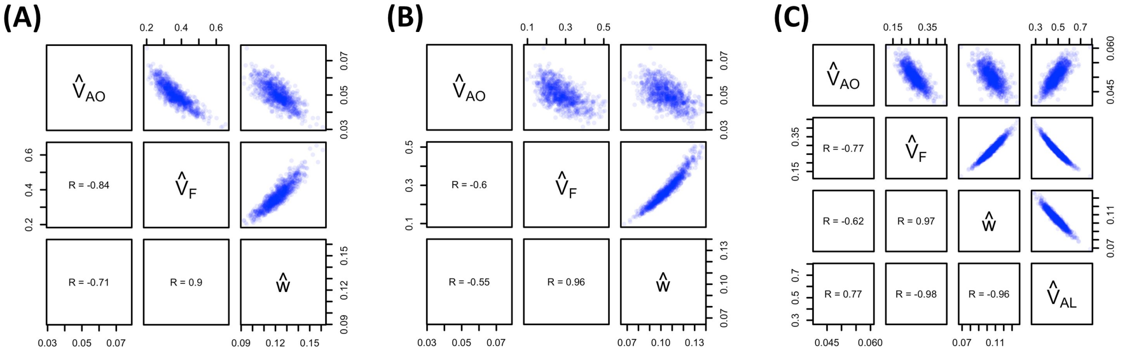

Suppl Figure 4. Scatter plots between estimates. Estimates are from $1K$ simulations where $r^2_{PGS,t0} = .05$, $r_{mate} = 0.25$, and AM is at disequilibrium. (A) shows scatter plots between estimates from model 1d. (B) shows scatter plots between estimates from model 2d-NP. (C) shows scatter plots between estimates from model 2d.

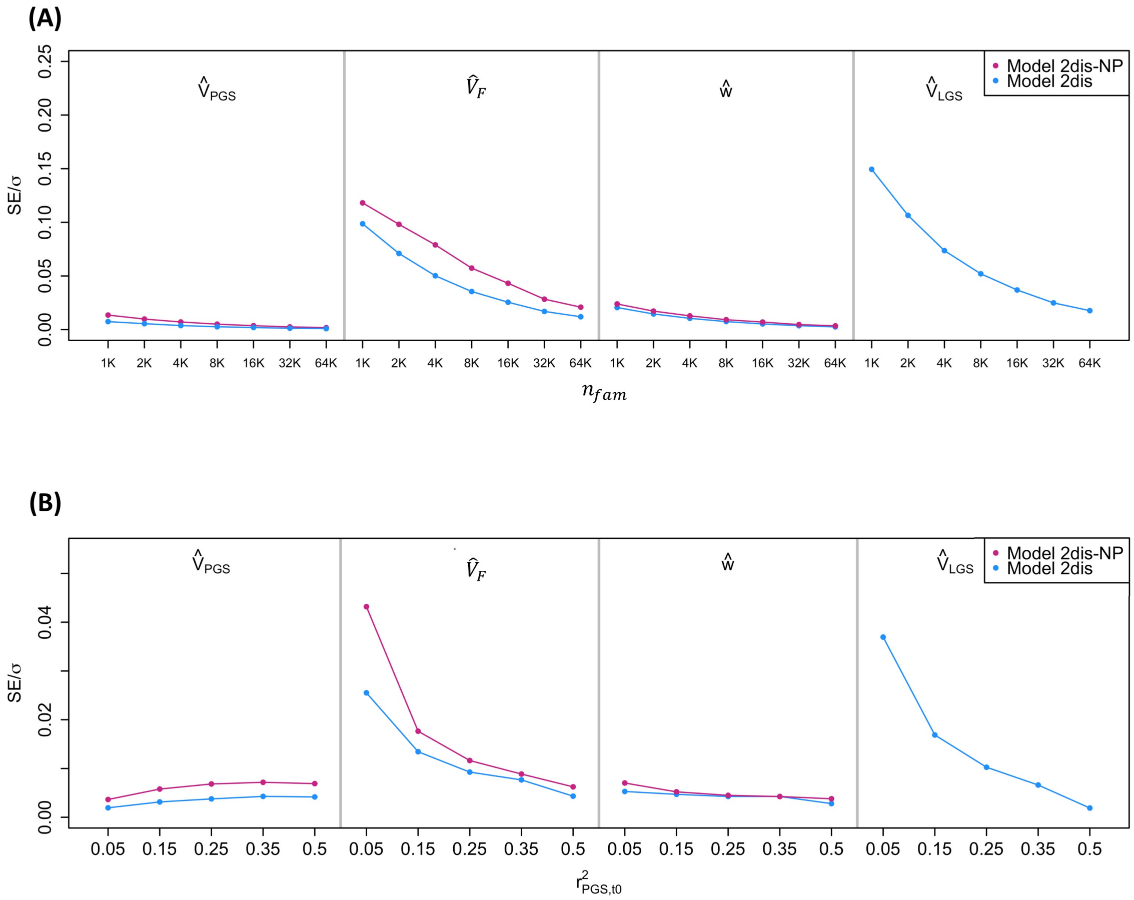

Suppl Figure 5. The standard errors (SE's) of standardized estimates from Models 2e and 2e-NP (A) as a function of n_{fam} when $r^2_{PGS,t0} = .05$ and (B) as a function of $r^2_{PGS,t0}$ when $n_{fam} = 16K$. Estimates are from $1K$ simulations where $r_{mate} = 0.25$ and AM is at disequilibrium.
